# The Gluten-Free Diet for Celiac Disease and Beyond

**DOI:** 10.3390/nu13113993

**Published:** 2021-11-09

**Authors:** Bara Aljada, Ahmed Zohni, Wael El-Matary

**Affiliations:** Section of Pediatric Gastroenterology, Department of Pediatrics and Child Health, Max Rady College of Medicine, University of Manitoba, Winnipeg, MB R3A 1S1, Canada; aljadab@myumanitoba.ca (B.A.); zohniah@gmail.com (A.Z.)

**Keywords:** celiac disease, gluten, gluten-free diet

## Abstract

The gluten-free diet (GFD) has gained popularity beyond its main medical indication as the treatment for gluten-induced immune-mediated disorders such as celiac disease (CD), dermatitis herpetiformis, gluten ataxia, wheat allergy, and non-celiac gluten sensitivity. However, the diet carries some disadvantages such as elevated costs, nutritional deficiencies, and social and psychological barriers. The present work aims to review indications, proven benefits, and adverse events of a gluten-free diet. Close follow-up with patients following the diet is recommended. More data is needed to assess the effectiveness of the diet in managing mental and cognitive disorders and to establish a connection between the brain and gluten.

## 1. Introduction

Wheat is responsible for 20% of global caloric consumption, making it amongst the most valuable crops worldwide. Due to its versatility, wheat can be incorporated into various foods such as bread, pasta, cereals, and baked goods, which has propelled this crop into a staple food across the temperate world [1]. Despite its traditional view as a nutritious source containing proteins, vitamins, and minerals, concerns have been raised towards a specific component of wheat called gluten. As an ingredient, gluten consumption dates back to 6th-century Chinese cuisine, where its popularity grew amongst Buddhists who used gluten as a substitute for meat. Jia Sixie’s *Qimin Yaoshu*, a Chinese agricultural encyclopedia written in 544 CE, mentions the use of gluten in noodles called bótuō. References of gluten in Western literature appear much later. Bartolomeo Beccari authored *De Frumento*, an Italian treatise on wheat, in 1745, which documented the extraction of gluten from wheat flour. In 1803, John Imson defined gluten in the English language in *Elements of Science and Art* [2]. The industrial revolution played a prominent role in the rising popularity of wheat as a staple food in the Western diet. Over this time, wheat was inexpensively milled in large quantities and quickly distributed using the developing railroad systems [3,4]. Western popularity of wheat also rose during the Great Depression and World War II, when wheat-containing products, such as bread and pasta, served as cheaper substitutes of rationed foods such as dairy and meat [5,6]. Today, global wheat consumption has increased at a faster rate than all other cereals [7]. As a result, there is increasing attention towards the health effects of gluten.

## 2. Gluten and Celiac Disease

Gluten is a mixture of water-insoluble prolamin proteins. The prolamins, a complex group of alcohol-soluble lectins, constitute the significant seed proteins in cereals. They comprise about 80% of the starch endosperm storage proteins in mature cereal grains [8] and are yet to be found in other parts of the grain [9]. The most abundant gluten prolamins (called gliadin and glutenin) are predominantly found in wheat. However, prolamins can be found in different cereal species under specific names, such as in barley (called hordeins), rye (secalins), oats (avenins), and other closely related grains although each has different molecular properties [10]. Gliadins comprise four significant alcohol-soluble monomers that collectively allow the gluten to elongate while providing intermolecular binding sites. The α-helices and β-sheets of α/β- and γ-gliadins allow for hydrogen and disulfide bonding, whereas ω-gliadins are composed of β-turns and have no α-helices or β-sheets [11]. In contrast, glutenins are alcohol-insoluble polymers that contribute to the flexibility and stability of gluten. When flour and water are mixed, a thiol group from glutenin interacts with disulfide bonds in gliadin, resulting in a shift towards intermolecular disulfide bonds [12]. The high concentration of glutamine amino acids results in many inter-chain hydrogen bonds that collectively provide strength [11,12]. In addition, gluten’s high proline content alters the protein structure to provide elasticity [11]. 

Gluten is infamous for its role in celiac disease (CD). This autoimmune condition affects 1% of the population and leads to a reversible inflammatory process in small bowel mucosa with acute repercussions such as diarrhea, constipation, bloating, nausea, and vomiting [13,14,15]. Long-term consequences of mucosal damage and inflammation include malabsorption of nutrients such as calcium, vitamin D [16], iron [17], vitamin B12, folic acid, and zinc [18], leading to debilitating consequences such as osteoporosis, anemia, and stunted growth [19]. The clinical presentation of CD can vary depending on age. The classic presentation in pediatric patients includes malnutrition, failure to thrive, abdominal pain, and distension. In contrast, adults commonly present with gastrointestinal symptoms but with less severity [20], with most patients experiencing severe diarrhea [21]. 

Calcium and vitamin D absorption is of particular concern in the growth and development of pediatric patients with CD. Several factors influence bone mineral density, including inflammation from chronic disease, diet, absorption in the duodenum, and metabolism [22,23]. In patients with CD, mucosal damage of the small bowel impairs calcium and vitamin D absorption, leading to impaired bone health. Whereas vitamin D is involved in the hormonal regulation of bone remodeling and calcium absorption [24], calcium serves a structural role in bones as a component of hydroxyapatite [25]. Pediatric patients with CD are at risk of short stature and constitutional delay of puberty. One study [26] found CD in 2–8% of children with short stature and no gastrointestinal symptoms. After ruling out endocrine causes for short stature, the same study found that the proportion of CD increased to 19–59%. When using a growth chart, pediatric patients with CD typically demonstrate a decline in both weight and stature velocity, crossing several percentile lines in both categories [27]. In addition, Ludvigsson et al. [28] found that patients with CD are at increased risk of subsequent hip fracture and fracture of any kind, independent of age or sex. A lower bone mineral density is one theory for the observed fracture risk, specifically in the femoral neck region, which Melton et al. [29] determined to be the strongest predictor of future hip fracture. Kemppainen et al. [30] supported this finding after they determined that patients had significantly lower bone mineral density at the lumbar spine and femoral neck, with over 64% of men and 71% of female patients presenting with low calcifediol, a form of vitamin D produced in the liver. 

The pathophysiology of CD involves a complex interplay between patients’ genetics and environment [31,32] that leads to an inappropriate immune response. In turn, the maladaptive response can cause enterocyte destruction and subsequent villous atrophy [20]. Once consumed, gluten’s glutamine and proline components prevent complete hydrolyzation of the immunoreactive epitope, producing peptides longer than ten amino acids in length [33]. Most notably, 13-, 19-, and 33-mer peptides are associated with the inflammatory reaction seen in CD [34,35]. In addition, gliadin prolamin upregulates the production of the intestinal peptide zonulin, which increases the permeability of tight junctions in the intestines. Several studies have shown increased levels of zonulin in patients with CD, making it a leading culprit in the pathogenesis of the disease [36,37]. In turn, these changes allow increased paracellular and transcellular peptide transport into the lamina propria [38]. Once in the gut mucosa, tissue transglutaminase (tTG) recognizes the glutamine and proline components, resulting in a series of deamidation and transamidation reactions that increases peptide affinity to antigen-presenting major histocompatibility complex class two (MHC II) molecules [20,39]. One study found human leukocyte antigens (HLA)-DQ2 and DQ8 present in 98.4% of patients with CD and a presence of 89.6% in their families, suggesting a genetic component to the disease [40]. Antigen-presenting cells, such as dendritic cells, present the peptides to gluten-specific T cells, triggering both the innate and adaptive immune response. The innate response releases interleukin (IL)-15, leading to the destruction of gut epithelial cells by CD8+ (cytotoxic) T-lymphocytes [41]. The role of IL-17 in the pathogenesis of CD is still under investigation. Scaleia et al. [42] found lower levels of IL-17-producing T cells in the intra-epithelial lymphocyte (IEL) compartment of CD patients. They speculate that these changes negatively affect the homeostasis of the mucosal barrier while contributing to the altered permeability of the gut mucosa. In addition, the adaptive response generates inflammatory cytokines, activating either interferon-gamma (IFN-γ) producing T helper (Th)1 cells or Th2 cells that promote B-lymphocyte development into plasma cells. In turn, plasma cells produce anti-gliadin and anti-tissue-transglutaminase antibodies [43]. The effects of gluten on the gut mucosa of susceptible individuals vary but can include gut inflammation, villous atrophy, crypt hyperplasia, and CD4+ and CD8+ T-cell lymphocytic invasion of the intraepithelial tissue [44]. When studying the histopathological effects of CD and response to treatment, clinicians have traditionally used the Marsh-Oberhuber classification system (Table 1), which grades biopsies of the intestinal mucosa into four categories. A diagnosis of CD is reserved for Marsh 2&3 biopsies, which show increased IELs, crypt hyperplasia, and villous atrophy. Marsh 3 can be divided into three subgroups based on the degree of villous atrophy [45].

## 3. Gluten-Free Diet for Celiac Disease

A lifetime gluten-free diet (GFD) is the treatment for individuals with CD [48]. Continuing to ingest gluten can exacerbate clinical symptoms, further intestinal damage, and increase the risk of future cancers, including small intestinal adenocarcinoma, esophageal cancer, melanoma, and non-Hodgkin’s lymphoma [49]. For best results, this diet involves complete removal of gluten-containing foods from one’s diet, including gluten proteins in wheat (gliadin), barley (hordeins), rye (secalins), oats (avenins), and other closely related grains. Due to such dietary cutbacks, individuals on a GFD are encouraged to incorporate other nutritious food sources such as fruits, vegetables, fish, meat, and gluten-free products. Over the years, scientific discovery, aggressive marketing, and media coverage of the benefits of a GFD have pushed food companies to produce more gluten-free options. As a result, 2016 saw over $15.5 billion in retail sales of gluten-free foods, more than double 2011 figures [50]. The marked increase in gluten-free substitutes allows CD patients to reproduce the dietary habits and patterns of the general population [51]. To support consumers following a GFD, the Food and Drug Administration (FDA) passed a gluten-free labeling rule that outlined the legal requirements for labeling a product “gluten-free”, “free of gluten”, “without gluten”, or “no gluten”. A gluten-free product is defined as having <20 ppm of gluten while considering possible contamination during product creation [52]. In addition, local organic food stores commonly sell gluten-free products such as bread and pasta, albeit at a slightly higher cost and with a different taste than their gluten-containing counterparts.

### 3.1. Efficacy of Gluten-Free Diet in Celiac Disease

There has been extensive research on the efficacy of the GFD. A strict GFD can restore the histology of the small bowel architecture in 95% of children within two years [53], whereas 34% and 66% of adult patients experience mucosal recovery after two and five years, respectively [54]. However, some data show incomplete recovery in older patients (between 30 and 60 years) and no statistically significant recovery in individuals older than 60 years [55]. With small bowel recovery, a GFD can also improve symptoms of malabsorption, including diarrhea, steatorrhea, and weight loss. In addition, several studies have demonstrated significant improvement in bone mineral density after one year of the diet [56,57,58], although complete reversal of osteopenia could not be observed [59]. Soliman et al. [60] found that pediatric patients on a GFD for two years demonstrate average growth in height and weight compared to age-matched controls, with significant catch-up growth (increase in percentile position on a growth curve) in some patients. When comparing the efficacy of GFD between patients with mild enteropathy and those with villous atrophy, Kurppa et al. established that the GFD has similar outcomes in mucosal architecture recovery, reduction of intestinal mucosal inflammation, antibody concentrations, and symptom improvement [61]. Another study examining the GFD in patients with borderline enteropathy that does not meet the criteria of CD demonstrated restoration of mucosal structure and marked improvement in clinical symptoms within 8–12 months of adhering to the diet compared to controls [62].

### 3.2. Skepticism of the Gluten-Free Diet

Despite the extensive literature on the GFD, questions and skepticism remain. Even with careful preparation and storage of gluten-free food, the likelihood of cross-contamination has raised questions about the effects of chronic low-dose gluten exposure [63]. Therefore, the focus towards the GFD has shifted from the absolute removal of gluten from one’s diet to limiting gluten intake below a specific threshold yet to be determined [64,65]. To identify the levels of safe gluten exposure, Akobeng and Thomas [66] reviewed thirty-five studies and found that gluten tolerability differed across studies and among study participants. While some patients had no histological abnormalities on a diet containing an average of 36 mg of gluten per day, others developed mucosal changes after only consuming 10 mg per day. They concluded that a daily intake of less than 10 mg is “unlikely to cause significant histological abnormalities.” In comparison, definite mucosal changes were observed with daily intakes of 100 mg and 500 mg, respectively [67]. Taken all together, one may conclude that achieving a conclusive threshold that could result in mucosal changes in 100% of patients with CD is unlikely to occur, although a daily intake of less than 10 mg is likely to produce the safest results. Skepticism has also been raised about the GFD’s ability to completely reverse abnormal changes in the gut mucosa. Gluten activation of the immune system has been shown to produce changes in the intra-epithelial lymphocyte compartment (IEL) and is associated with increased γ/δ IELs. Recent cell sequencing work found high levels of γ/δ IELs in histologically normal-appearing tissue, suggesting that some changes persist following a GFD [68].

### 3.3. Challenges of a Gluten-Free Diet

Given that gluten-containing food represents staple dietary components in many households worldwide, a GFD represents a dramatic lifestyle change that can pose many challenges. The threat of cross-contamination is a daily issue for individuals on a GFD. Sharing cupboards, countertops, and kitchen appliances with individuals who do not follow a GFD present possible contamination opportunities that impair the diet’s success. For increased safety, meals should be prepared and stored away from non-gluten-free food. A similar concern extends to eating at restaurants, food courts, and food stands. Although individuals may face difficulty finding gluten-free options, restaurants are increasing their gluten-free options due to the rising popularity of the GFD. Of note, one study found that 32% of gluten-free labeled restaurant food tested positive for gluten, with gluten-free pizza and pasta being the most likely culprits [69]. Processed foods made from gluten-containing ingredients represent another area of concern. Potentially hidden sources of gluten include certain soups, processed meat, French fries, seasonings, and beer. Although the degree of susceptibility to gluten-containing food varies between individuals, one study suggests a safe gluten contamination cutoff of 100 ppm (1/4 mg/kg) in gluten-free foods [70]. Therefore, eating foods that have a gluten-free label is generally a safe option for avoiding gluten-contaminated food. Finally, adhering to a GFD can be costly. In one study, gluten-free products were 242% more expensive than their gluten-containing counterparts in the same food group [71]. Several studies echoed this sentiment, demonstrating lower availability and higher cost of gluten-free foods [72,73]. However, despite these challenges, a prospective study by Mustalahti et al. [74] found declining symptoms and a significantly improved quality of life in patients with CD on a GFD, suggesting that the diet was not particularly distressing for the majority of patients.

### 3.4. How to Monitor a Gluten-Free Diet for Celiac Disease

**Figure 1:**
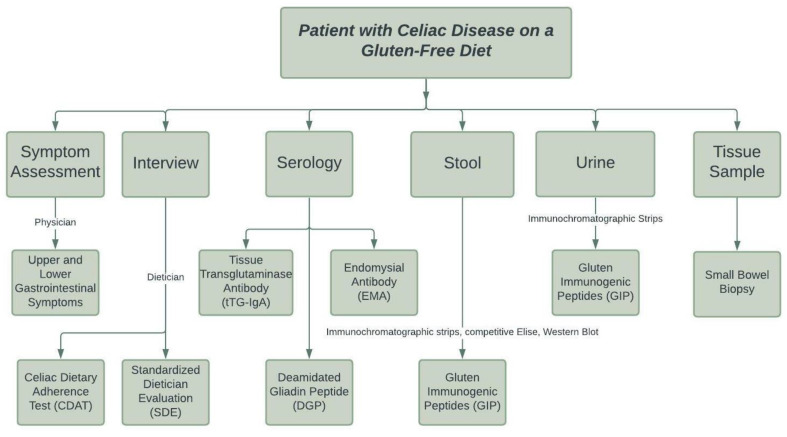
Methods for monitoring adherence to gluten-free diet.

Strict adherence to a gluten-free diet is the only recommended treatment for CD [75]. As such, one may suggest that newly diagnosed and symptomatic patients require more frequent assessment, especially as the gut mucosa is undergoing repair and clinical symptoms are improving. Several studies have investigated when patients should be followed up after initiating a GFD and with whom, given that there is no clear consensus. In a study examining patient preferences towards follow-up, most preferred to be seen by a dietician (with a physician available if needed), and 67% of respondents preferring annual appointments [76]. Kurppa et al. [77] found that follow-up by primary care physicians was just as successful as a follow-up in tertiary centers, with average GFD adherence rates at 88%. Current guidelines recommend routine blood tests at each follow-up visit, including checking for intestinal absorption with a complete blood count, serum calcium, ferritin, vitamin B12, and alkaline phosphate. In addition, thyroid function tests such as thyroid-stimulating hormone and thyroid hormone should be checked to screen for other autoimmune conditions, alongside liver function tests such as aspartate aminotransferase and alanine aminotransferase levels to monitor for autoimmune liver disease [44]. While there are no strong recommendations towards a particular monitoring tool, there are several methods for monitoring gluten-free diet adherence and efficacy in CD, including symptom assessment, dietetic interview, serology, stool and urine markers, and small bowel biopsy (Figure 1).

#### 3.4.1. Symptom Assessment

The first step of monitoring the GFD in patients with CD is to identify ongoing symptoms and their severity. One study [78] found that upper gastrointestinal symptoms disappear first, while lower gastrointestinal tract symptoms, such as constipation, remain unchanged when re-evaluated 12–28 months after beginning a GFD. Abdominal bloating (51.3%), abdominal pain (45.9%), and constipation (29.7%) represent the most common symptoms at follow-up. The strongest positive predictors for ongoing symptoms at re-evaluation include experiencing symptoms for five years or more before diagnosis (OR 5.3, 95% CI 1.3 to 21.8) and having constipation at the time of diagnosis (OR 7.4, 95% CI 1.3 to 42). However, Rubio-Tapia et al. [54] found that clinical response to a GFD was an inaccurate marker for mucosal repair. Additionally, 62% of patients who experienced a clinical response to a GFD have continued mucosal damage at their follow-up biopsy, although symptomatic patients do not present with more severe histological lesions than asymptomatic patients [78]. Lahdeaho et al. [70] supported the limited utility of clinical response as a monitoring tool when they found that 22% of patients with significant small bowel damage had no symptoms. Nonetheless, symptomatic improvement is a potential motivator for the continued adherence to a GFD and serves as a limited tool for monitoring the disease.

#### 3.4.2. Dietetic Interview

A second option for monitoring the GFD is a dietetic interview conducted by a trained dietician or physician. There are various questionnaires available in a variety of languages that assess self-reported compliance with the GFD. The results from these surveys are often combined with visual analog scales that contain unmarked lines with anchor statements such as ‘I never follow my diet’ and ‘I always follow my diet’ at the boundaries [44,79]. Currently, the Standardized Dietician Evaluation (SDE) is the gold-standard interview format for assessing adherence to the GFD. A trained dietician conducts this interview, consisting of three main parts from which answers are graded according to a 6-point Likert scale. First, the dietician analyzes the patient’s diet over twenty-four hours or three days. The patient then participates in a food-label quiz to determine which ingredients and additives are likely to contain gluten from a list of twenty-eight. Finally, the patient is assessed on their ability to check the labels of medicines, supplements, and cosmetics for gluten [80]. The Celiac Dietary Adherence Test (CDAT) is another popular screening tool. Developed by gastroenterologists, dieticians, psychologists, and celiac patients, this tool grades participants’ answers to seven questions regarding their knowledge, opinions, and adherence to a GFD on a 5-point Likert scale [81]. Although the CDAT is highly correlated with the SDE [80], the SDE shows a stronger correlation with serological titers and duodenal biopsies [81]. Subjectivity, fear of judgment, and under-reporting of gluten consumption represent significant limitations to the interview format [82].

#### 3.4.3. Serology

Serological testing for antibodies associated with CD is another option for monitoring the GFD. Elevated levels of tissue transglutaminase antibodies (tTG-IgA), endomysial antibodies (EMA), and deamidated gliadin peptide (DGP) antibodies can indicate poor adherence to or efficacy of a GFD. Testing for tTG-IgA is a first-line diagnostic tool in the workup of CD with sensitivity and specificity levels above 95% [83,84]. Relative to other markers, the combination of high sensitivity, functionality, and cheaper costs of tTG-IgA make it a preferred choice for initial serological testing. Positive tTG-IgA results are often followed up by confirmatory EMA testing, which shows a higher specificity (99.0–100%) for CD [85,86]. Some studies have shown rising serum EMA levels before the appearance of villous atrophy, making it a potential early marker in CD [61,87]. Limitations of this marker include higher operating costs and less objective results due to the use of labor-intensive, resource-demanding, and operator-dependent immunofluorescence [88]. Testing for DGP is a newer technique for CD, although it poses a lower sensitivity (88%) and specificity (94%) in the general population than the markers mentioned earlier [89]. Nonetheless, new evidence suggests that testing for DGP is of better use in pediatric patients for diagnosing CD and monitoring a GFD. In an investigation of forty children less than two years of age with features of chronic enteropathy, Barbato et al. found eleven patients with normal tTG, and EMA titers with elevated DGP and endoscopic changes consistent with CD [90]. In addition, Monzani et al. demonstrated that testing for DGP IgA and IgG in children had a sensitivity of 100% for screening for CD and was 52% more sensitive than tTG for monitoring GFD adherence [91]. A study by Liu et al. found that DGP levels normalized faster than tTg in children following initiation of a GFD, making DGP a possible early marker of a response to a GFD [92]. However, despite these antibodies’ reported high specificity and sensitivity for diagnosing CD, serology has several limitations in its use as a marker for GFD efficacy, especially as it relates to mucosal repair. Serological markers represent the body’s immune response to the disease and are not directly correlated with intestinal damage. A meta-analysis on the sensitivity and specificity of tTG IgA and EMA IgA assays determined that both serological markers had a poor correlation with mucosal damage in celiac patients undergoing a follow-up biopsy while on a GFD. In patients with villous atrophy (Marsh 3), tTG IgA had a sensitivity of 0.50 (95% CI 0.41–0.60) and a specificity of 0.83 (95% CI 0.79–0.87), while EMA IgA had a sensitivity of 0.45 (95% CI 0.34–0.57) and specificity of 0.91 (95% CI 0.87–0.94). Although a positive test result is a good indicator of persistent villous atrophy, most patients with mucosal damage will have normal antibody titers while on the GFD, making serology an unreliable marker for following mucosal repair and monitoring adherence [93].

#### 3.4.4. Stool and Urine Markers

Clinicians can also use stool and urine markers for monitoring GFD. Specific gluten peptides, such as the immunotoxic 33-mer peptide, are resistant to gastrointestinal degradation. In one study, over 30% of 33-mer peptides resisted hydrolysis in vitro simulated gastrointestinal digestion [94]. The degree of immunotoxic peptide absorption and excretion varies among individuals and can be influenced by differences in the gut microbiome and diet [95]. Some peptides are subsequently excreted in feces and can be detected by immunochromatographic strips, competitive ELISA, and Western blot [94,96]. In turn, detection of gliadin peptides in the stool can be used as evidence of gluten consumption and as a non-invasive marker of compliance with a GFD [96,97,98]. Whereas immunochromatographic strips are more likely to be used as clinical standard assays in point-of-care settings, ELISA is more likely to be used for more detailed quantification of gluten exposure when monitoring the efficacy of a GFD [99,100]. Comino et al. [94] found that ingestion of 50 mg of gluten was enough for detection in stool samples and that levels of gluten consumption were “roughly” correlated with gluten excretion 2–4 days after ingestion. The study concluded that the non-invasive nature of the immunologic tests could be used to monitor short-term adherence to GFD, involuntary gluten consumption from contaminated food, and for assessing the effectiveness of novel treatments for CD such as enzymatic therapies designed to destroy toxic gluten peptides. Another multicenter study [100] examined the use of ELISA to detect immunogenic gluten peptides (GIP) in patients on a GFD for at least one year and to compare the assay to other GFD monitoring tools. Researchers found that 30% of patients on a GFD had detectable GIP in their stools, suggesting they were either non-compliant with the GFD or involuntarily consuming contaminated food. The presence of GIP was strongly associated with symptoms associated with gluten exposure, with up to two-thirds of patients unresponsive to a GFD having detectable GIP on ELISA. In contrast, stand-alone use of dietary questionnaires and serum anti-tTG antibody levels revealed non-compliance in 18% of the same patients. The same study found no significant association between stool GIP and dietary questionnaires or serum anti-tTG antibody levels. 

Researchers have also explored the use of urine samples as a monitoring tool for compliance and efficacy of a GFD. Moreno et al. [101] found that ingestion of greater than 25 mg of gluten results in urine GIP that are detectable on immunochromatographic strips as early as four hours after ingestion and remain detectable in the urine for up to 48 h. In addition, GIP levels were positively correlated with the level of gluten intake. Moreover, 89% of patients with CD and no intestinal mucosa damage on duodenal biopsy had no detectable GIP in their urine. Consequently, all patients with incomplete recovery of the mucosa had quantifiable GIP. Various factors can influence GIP concentration in the urine, including diet, daily liquid intake, weight, and gut microbiota. 

#### 3.4.5. Small Bowel Biopsy and Pathology

At present, assessing small bowel pathology is the most accurate method for monitoring mucosal recovery in patients on a GFD. Several studies have shown mucosal damage on biopsy in patients with normal serology and clinical response to a GFD [54,102]. When performing a biopsy, multiple small bowel samples are collected given the patchy nature of histological abnormalities in CD and the declining sensitivity of biopsies for CD when less than four samples are taken [103,104]. Current guidelines give a strong recommendation, backed by a high level of evidence, for at least one duodenal bulb biopsy and at least four biopsies of the distal duodenum [104,105]. Pathologists look for the presence of crypt elongation and villous atrophy, the density of IELs, and the crypt-villous ratio while classifying the specimen according to the Marsh-Oberhuber scale [105]. Rubio-Tapia et al. [54] examined the rate of mucosal recovery, defined as a villous to crypt ratio of 3 to 1, at the first follow-up biopsy for adult patients on a GFD. Moreover, 35% of patients on a GFD receiving a biopsy within two years of starting the diet showed mucosal recovery, whereas 43% showed mucosal recovery when the first biopsy was taken between two to five years after initial diagnosis. Histological improvement, characterized by an increase of villous to crypt ratio ≥2.0 points relative to baseline, was observed in 45% of patients at the first follow-up biopsy. In addition, the average recovery time for mucosal repair was determined to be three years after starting a GFD. Although complete histological recovery is not universally achieved on a GFD, various studies suggest that mucosal healing can be seen in 57–76% of patients [44]. Compared to adults, pediatric patients s89howed a better response to the GFD, with up to 95% showing mucosal recovery within two years [53]. However, intestinal biopsies are more invasive, expensive, and unreasonable for monitoring every patient with CD than other monitoring tools. For this reason, the American College of Gastroenterology [104] gives a strong recommendation for long-term follow-up of a GFD based on history and serology alone. They further suggest that biopsies should be reserved for patients showing inadequate clinical response or relapse in symptoms while on a GFD.

## 4. Gluten-Free Diet for Other Health Problems

**Figure 2:**
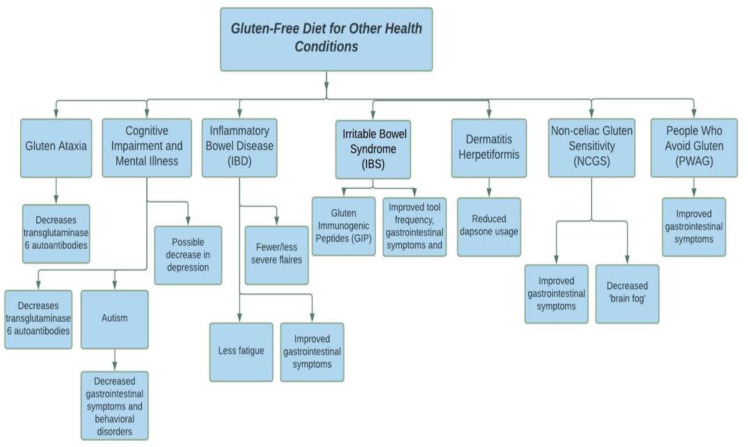
The GFD for health conditions other than celiac disease.

The gluten-free diet is recognized as the standard protocol for patients diagnosed with CD. However, the diet has recently gone mainstream, and individuals excluded from the CD diagnosis now make up most adherents. Chuong and colleagues [106] found that between 2009 and 2014, the prevalence of CD in the American population remained constant (0.7%) while the demographic of people who avoid gluten (PWAG) grew from 0.5% to 1.7%. Since the gluten-free diet is no longer a niche treatment for a select diagnosis and is now utilized more broadly by the general population, many studies have analyzed the benefits of the diet. Beyond patients with CD, the gluten-free diet is also recognized in the treatment of gluten ataxia, dermatitis herpetiformis, cognitive impairment, inflammatory bowel disease and irritable bowel syndrome, dermatitis herpetiformis, and non-celiac gluten sensitivity (Figure 2).

### 4.1. Gluten Ataxia

Gluten ataxia is an immune-mediated disease wherein ingestion of gluten causes the body’s immune system to attack the nervous system tissue, specifically the cerebellum. Transglutaminase 6 (TG6) autoantibodies are more abundant in patients with gluten ataxia and have become an efficient marker for diagnosing the condition, as demonstrated by Hadjivassiliou and colleagues [107]. These antibodies are suspected to be the primary mechanism through which neurological diseases develop in individuals with gluten sensitivities. A study by Dipper and colleagues demonstrated that patients placed on a GFD experienced a decrease in TG6 autoantibodies and a sustained normalization in those who continued to follow the diet [108], suggesting that a GFD can be used to contain symptoms of gluten ataxia. While the GFD has proven its efficacy in treating gluten ataxia and CD, much of its perceived benefits towards other health problems remain questionable.

### 4.2. Cognitive Impairment and Neurological and Mental Illnesses

Recent studies have shown that there may be a correlation between gluten sensitivity and neurological diseases. Since TG6 autoantibodies are known to attack the nervous system as an immune-mediated reaction to gluten ingestion, a link may exist between this mechanism and other neurological illnesses beyond gluten ataxia. A study conducted by Hadjivassiliou and colleagues [109] analyzed the serum levels of antigliadin antibodies in 147 neurological patients, of which 53 (25 ataxia, 20 peripheral neuropathy, 5 mononeuritis multiplex, 4 myopathy, 3 motor neuropathy, 2 myelopathy) had no known cause for their diagnosis despite full investigation. They were compared alongside a second group of 94 patients that had known causes for their diagnosis. Finally, 50 healthy blood donors were used as the third group. Results demonstrated that the first group had significantly higher positive serum anti-gliadin antibodies than the other groups (57%, 5%, and 12%, respectively). These data establish a strong correlation between gluten sensitivity and neurological illnesses. Finally, another neurological illness of concern as it pertains to the GFD is autism. Autism diagnosis has started to increase, with a diagnosis of 1 in 88 children [110]. Patients with autism have a higher prevalence of IgG antibodies to gliadin, the same antibodies associated with CD and gluten ataxia [111]. Since many children with autism have gastrointestinal symptoms, there seems to be a link between autism and gluten sensitivity. In a study conducted by Ghalichi and colleagues [112], 80 children with autism spectrum disorders (ASD) received either a GFD treatment (n = 40) or a regular diet treatment (n = 40); 53.9% of the children reported having gastrointestinal abnormalities. The ROME III questionnaire for evaluating gastrointestinal symptoms and Gilliam Autism Rating Scale 2 questionnaire (GARS-2) for assessing psychometric properties were used to evaluate the effects of the GFD versus the regular diet. Results demonstrated that children placed on the GFD experienced a significant decrease in both gastrointestinal symptoms (40.57% vs. 17.10%, *p* < 0.05) and behavioral disorders (80.03 ± 14.07 vs. 75.82 ± 15.37, *p* < 0.05). The children placed on the regular diet experienced an insignificant increase in both metrics. The research, however, is somewhat conflicting on this topic. Piwowarczyk and colleagues demonstrated that a GFD did not influence autistic symptoms, maladaptive behaviors, or intellectual abilities [113]. The relief in gastrointestinal symptoms in children with autism placed on a GFD is in accord with most of the literature, given that patients with elevated levels of IgG antibodies to gliadin tend to experience similar effects. However, the influence of the GFD on autistic symptoms and intellectual abilities is not well established.

Some evidence has emerged on the potential benefits of the gluten-free diet for depressive disorders, although the studies on this topic are scarce, and further investigations may be needed. Peters and colleagues [114] conducted a study that tested 22 patients with irritable bowel syndrome who had a negative CD diagnosis. The authors utilized a double-blind cross-over method which consisted of 3 days of one of 3 dietary challenges (diet supplemented with gluten, whey, and no supplement (placebo)) followed by a 3-day washout period before crossing-over. The mental state was assessed using the Spielberger State-Trait Personality Inventory (STPI), and results demonstrated that depression scores in the gluten group were higher than the placebo group (*M* = 2.03, 95% CI (0.55–3.51), *p* = 0.010). The whey ingestion group did not show significant differences in depression rates, cortisol secretion, or gastrointestinal symptoms. These results prompted the conclusion that a correlation could exist between depressive disorders and gluten ingestion. Another study conducted by Zylberberg and colleagues [115] found similar results in people who avoided gluten. Data from 22,274 participants of the 2009–2014 National Health and Nutrition Examination Survey compared depression, insomnia, quality of life variables, and psychotropic medication use in CD patients and people who avoid gluten to controls. The results obtained showed no increased odds of depression or sleep difficulty among CD patients. People who avoid gluten, however, had lower odds of depression compared to control after adjustments. The study calls for further investigation into the correlation between gluten exposure and depression. Since people who avoid gluten do so out of their conviction, they could be more health-conscious than the CD patients and control group without any formal diagnosis. Given that physical health is closely associated with mental health, there could be some confounding effects [116,117]. Moreover, schizophrenia is a particular mental health disease of interest when discussing the gluten-free diet. Some studies have shown that schizophrenic patients tend to have elevated anti-gliadin antibodies and transglutaminase 6 antibodies [115] despite not having a CD diagnosis. A review of articles conducted by Ergün, Urhan, and Ayer [118] found that symptoms of schizophrenia improved following the elimination of gluten from the diet. Another systematic review, conducted by Levinta and colleagues [119], searched different databases and found 9 studies relevant to gluten and schizophrenia; 6 of the studies demonstrated beneficial effects, namely decreased severity in symptoms and improved functioning. However, they found that only one of the studies was a randomized controlled trial, while seven were cross-over studies and one was an open-label pilot study. For this reason, the conclusions of the systematic review are limited. Nonetheless, there seems to be a connection between the consumption of gluten and schizophrenic disorders.

### 4.3. Inflammatory Bowel Disease and Irritable Bowel Syndrome

The GFD has also been utilized as a potential treatment for irritable bowel syndrome (IBS). Diarrhea-dominant irritable bowel syndrome (d-IBS) patients tend to experience symptom relief following the introduction of a gluten-free diet. In a study conducted by Wahnschaffe and colleagues, 60% of d-IBS patients positive for human leukocyte antigen (HLA)-DQ2 T-cell haplotypes, and CD-associated serum IgG had improved stool frequency. Moreover, gastrointestinal symptom scores returned to normal after 6 months of a gluten-free diet compared to 12% negative for these biomarkers [120]. While the patients with d-IBS were positive for CD biomarkers, these antibodies were not always collected. Therefore, the patients would be classified as having non-celiac gluten sensitivity. Another study conducted by Aziz et al. analyzed the effect of a 6-week gluten-free diet on patients with d-IBS (20 HLA-DQ2/8-positive and 21 HLA-DQ2/8-negative). Twenty-nine patients (71%) reported having their symptoms relieved following the completion of the trial [121]. These two studies demonstrate the potential benefits of the gluten-free diet for patients with d-IBS. Patients with inflammatory bowel disease (IBD) also appear to benefit from a GFD. Patients with CD are also more likely to have an IBD diagnosis than the general population [122]. Herfarth and colleagues [123] conducted a study analyzing the effects of a GFD on 1647 patients with IBD. CD and non-celiac gluten sensitivity were reported by 10 (0.6%) and 81 (4.9%) respondents, respectively; 314 participants reported having previously tried a GFD, and 135 reported current use of GFD (19.1% and 8.2%, respectively). Overall, 65.6% of all patients who attempted a GFD described improving their gastrointestinal symptoms, and 38.3% reported fewer or less severe IBD flares. Patients who were strict in GFD adherence also reported less fatigue. In addition, Lindberg and colleagues [124] compared the levels of IgG, IgA, and IgM antibodies to baker’s yeast (saccharomyces cerevisiae), yeast mannan, gliadin, ovalbumin, and beta-lactoglobulin in twins with IBD versus those of healthy controls. Results demonstrated that the twins with ulcerative colitis had elevated IgA antibodies to gliadin levels than the other twins and healthy controls. For these reasons, the GFD may be an effective symptom managing diet in patients with ulcerative colitis IBD.

### 4.4. Dermatitis Herpetiformis

Treatment of patients with dermatitis herpetiformis (DH) with a GFD has been demonstrated to be highly effective [125,126]. In a study conducted by Reunala and colleagues [125], 81 patients with DH were treated with a GFD and a standard diet (control); 93% of patients placed on a GFD were able to reduce their dosage of dapsone, an antibiotic used in the treatment of DH, versus 16% in the control group. In addition, 28% of the GFD group were able to eliminate the antibiotic without experiencing any symptom aggravation. Another study conducted by Lionel et al. [126] demonstrated similar results. Twenty-four patients with DH were treated with a GFD and 16 (80%) were able to reduce their dapsone usage. Ten of the patients were able to eliminate the antibiotic and were free of any skin lesions. These two studies provide satisfactory evidence demonstrating the efficacy of a GFD in the treatment of DH.

### 4.5. Non-Celiac Gluten Sensitivity (NCGS) and People Who Avoid Gluten

While the benefits of the GFD in treating CD and gluten ataxia are established in the literature, many studies have sought to investigate whether the diet is viable in treating other conditions. A biopsy is generally needed to diagnose a patient with CD, requiring a gluten-free diet for treatment. In recent times, however, patients who were excluded from a CD diagnosis but had IBS-like symptoms when exposed to gluten have been put under the non-celiac gluten sensitivity (NCGS) umbrella. Patients with NCGS tend to have normal small intestinal permeability and will experience IBS-like symptoms such as bloating, stomach pain, fatigue, rash, and discomfort upon consuming gluten. The scientific literature is not always clear when establishing a diagnosis for this condition as the overlap with irritable bowel syndrome is strong. Patients with NCGS do not express CD-related antibodies and are generally harder to diagnose as they do not have well-defined biomarkers. Still, there is strong evidence that supports the existence of this condition [127,128,129]. Theories have proposed that patients with NCGS may be sensitive to another component of wheat besides gluten, namely the amylase-trypsin inhibitors, which trigger a similar immune response as gluten [130]. Wheat germ agglutinin is another plant protein found in wheat that has been shown to trigger similar immune responses [131]. Thus, nonceliac gluten sensitivity patients may be more sensitive to wheat in general instead of specifically gluten, and the term non-celiac wheat sensitivity may describe the condition better. To complicate matters, a study conducted by Skodje and colleagues [132] found that NCGS patients experienced worsened symptoms following consumption of fructan but not gluten. Fructan is oligo, di, and monosaccharides that are often found in foods that also contain gluten. The double-blind cross-over challenge found that 59 self-diagnosed NCGS individuals following a gluten-free diet experienced more symptoms based on the Gastrointestinal Symptom Rating Scale Irritable Bowel Syndrome (GSRS-IBS) following ingestion of fructan than following ingestion of gluten. No significant differences were found between gluten and placebo or fructan and placebo. While NCGS patients may not be specifically sensitive to gluten, they could be sensitive to other factors that are generally found alongside gluten. Impairments in cognitive health have been observed in some patients with gluten sensitivity before treatment. Brain fog, which is in the spectrum of non-celiac gluten sensitivity (NCGS), refers to problems involving memory, attention, executive function, and cognitive processing speed. Patients with NCGS often report this condition, and a gluten-free diet has been observed to improve some of these symptoms after one year of adherence [133].

People who avoid gluten (PWAG) are a broader term that describes GFD adherents excluded from CD and non-celiac gluten sensitivity through rechallenge tests. PWAG make up the largest demographic of gluten adherents. PWAG generally tend to do so due to its perceived benefits. However, as mentioned below, the gluten-free diet does not come without its adverse outcomes [50]. Therefore, it is important to educate individuals who adhere to the GFD without any diagnosis about the potential risks, given that these individuals do not immediately require the diet.

## 5. Adverse Events of GFD

While the benefits of a GFD seem alluring, it is important to consider the risks associated with the regiment. Much of the studies conducted on its health complications appear inconclusive and even conflicting. One of the main concerns of the GFD is the lack of beneficiary whole grains consumed by adherents, which can be a factor in coronary heart disease [134,135,136,137]. Assessing this hypothesis, Lebwohl et al. [138] studied the development of coronary heart disease in 64,714 women in the Nurses’ Health Study and 45,303 men in the Health Professionals Follow-up Study. Food diaries were updated every 4 years from 1986 through 2010 and were used to assess the amount of gluten consumed. Results demonstrated an inverse relation between gluten intake and coronary heart disease risks. On the other hand, a systematic review conducted by Potter and colleagues [139] analyzed 27 articles on patients who adopted the GFD. Findings included increases in high-density lipoproteins, fasting glycemia, total cholesterol, and body mass index, although the increases in metrics were within a healthy range. The review did not find any increase in triglycerides, low-density lipoprotein, or blood pressure, prompting the conclusion that the GFD is not associated with coronary heart disease. Of note, only one of the articles had a control group and was limited by several confounders, so proper analysis is limited. Another analysis, conducted by Heikkilä and colleagues [140], found some support for the association of coronary heart disease with the GFD; however, they state that the evidence base was weak and had limitations. Finally, Kim and colleagues [141] demonstrated that the GFD was beneficial in waist circumference reduction and lowered BMI while maintaining that the diet was not associated with elevated cardiovascular disease risks. GFD followers, who were primarily women and were health-conscious, were found to have lower metabolic syndrome and lower cardiovascular disease risks, although the difference was not statistically significant. Overall, most studies have called for more research to examine this hypothesis, as no conclusive findings have been made. Many studies lean towards excluding the GFD as a factor in cardiovascular disease risk. While the literature seems inconclusive regarding the GFD and coronary heart disease, other adversities associated with the regiment are clearer. Recent evidence suggests that the diet may worsen the gut microbiota while having nutritional deficiencies in iron, calcium, and fiber [142,143,144,145]. The diet is also associated with a high cost due to the further processing required for gluten-free alternatives [146]. Finally, some research has raised concerns about the negative social and psychological impacts that many GFD adherents experience, mainly due to the diet’s restrictive nature [147,148].

### 5.1. Gluten and the Gut Microbiome

The importance of healthy gut microbiota in maintaining good health is becoming increasingly evident in the literature. The human gut contains two main phyla of bacteria, Bacteroidetes and Firmicutes. The role of these bacteria is highly diverse and includes the metabolism of nutrients consumed by the host, xenobiotic and drug metabolism, maintenance of structural integrity of the gut mucosal barrier, protection against pathogens, and immunomodulation [149]. Diet can also affect the health of gut flora, along with other factors such as birthing method (vaginal canal vs. cesarean) [150] and use of antibiotics [151]. In their study, David and colleagues [152] demonstrated that changes in diet rapidly influence the composition of the gut flora. Thus, it is important to consider the effects of gluten consumption and restriction on the health of the bacteria found in the host’s gut. Golfetto and colleagues [153] conducted a study on 42 healthy subjects and 14 patients with CD to analyze the health of their gut bacteria. The study found that patients with CD had an imbalance in their intestinal microbiota despite being on a gluten-free diet. It is unknown whether the patients with CD had this imbalance before adhering to a GFD or whether they developed it later. Regardless, the diet does seem to cause this imbalance to persist. It is important to address these imbalances as they can cause the gastrointestinal symptoms patients may experience when consuming gluten [154]. In another study conducted by Palma and colleagues [155], 10 healthy subjects were introduced to a GFD, and their gut microbiota was monitored for a month. Results demonstrated a reduction in beneficial gut bacteria, raising concerns over the potential risks of the GFD. If gluten exclusion from the diet results in imbalances and a reduction in healthy gut flora, it is important to address those issues by providing support. Probiotic supplements are of particular interest as they can balance the gut flora and provide it with the nutrients it needs to remain healthy [145,156,157].

### 5.2. Nutritional Deficiencies

Concerns have been raised about the nutritional quality of GFD. As the diet has gained popularity from media coverage and celebrity promotion, many people have adopted the regiment despite having no diagnosed CD. For these individuals, gluten avoidance may cause nutritional deficiencies which could otherwise be prevented. For example, abnormal intake of vitamin D has been linked to the GFD. In a study conducted by Deora and colleagues [142], the medical records of 140 children with CD were assessed, and 70% of these children had vitamin D deficiency at the time of diagnosis. After 6 months of GFD adherence, these children found a slight improvement in their vitamin D uptake, although levels remained abnormal. Given that vitamin D is crucial to intestinal uptake of minerals, it is important to address this issue through supplementation and dietary adjustments. The diet also presents other deficiency concerns beyond vitamin D. In 2005, a survey conducted by Thompson and colleagues [143] in patients with CD found that women had a mean average intake of 46%, 44%, and 31% of their daily fiber, iron, and calcium intake requirements. In men, the values were 88%, 100%, and 63%, respectively. These results demonstrated that women who adhered to the GFD might be at risk of nutritional deficiencies, even more so than men. In addition, a systematic review conducted by Di Nardo et al. [158] found that all children, regardless of whether they were diagnosed with CD or not, were at risk of nutritional deficiencies (insufficient fiber, iron, vitamin D, and calcium). Moreover, children with CD following a GFD had inadequate folate, magnesium, and zinc consumption, and higher consumption of high glycemic index foods. The paper suggested the need for therapeutic protocols to include education about these deficiencies so patients can ensure their diet is complete. Another disadvantage of the GFD is the potentially elevated level of lipid and protein consumption. Mariani and colleagues [159], in a survey analyzing the 3-day alimentary intake of 47 adolescents with CD, found that strict adherents to the GFD had increased intakes in protein and lipid, as well as a more significant prevalence of obesity (72% vs. 47% in control). These results are expected, as gluten tends to occur in carbohydrate-rich foods naturally and not protein- or lipid-rich foods. It is important to note that the quality of the lipids and proteins should be of concern and not necessarily the amount consumed. An analysis of gluten-free biscuits by Caponio et al. [160] found that they contained a sizeable mean amount of low-quality oleic trans-isomer fats (9.39%). Much of the literature suggests mitigating this negative side of the GFD by consuming more naturally gluten-free products and avoiding processed gluten-free alternatives as they do not seem to provide many nutritional benefits. It is important to note that many of the studies conducted on the deficiencies of the GFD have studied CD patients who suffer from gut inflammation and lack proper nutrient uptake. This may be a confounding factor as the results pertain to individuals affected by the disease and may not apply to those without CD. With that in mind, the GFD seems to have some nutritional disadvantages, namely deficiencies in vitamin D, iron, calcium, folate, and dietary fibers, and a higher amount of low-quality lipids found in some gluten-free alternatives [142,143,144]. Whether afflicted with CD or not, adherents of the diet should ensure that they reach daily recommended requirements for all minerals listed above. Avoiding processed gluten-free alternatives and eating naturally-occurring gluten-free foods high in iron, such as meats, fish, and green vegetables, is a recommended solution to this dietary problem associated with the diet [158].

### 5.3. Cost

Cost is another challenge associated with the GFD. Most products that naturally contain gluten, such as pasta and bread, require little to no processing to produce. Gluten-containing foods have been around for thousands of years and are found in many popular recipes. Bread, for example, is a staple in many dishes and diets across the world. Grains generally tend to be cheap to produce and grow in a wide range of climates, making them ideal for consumption. As these tend to contain gluten naturally, further processing is required to remove the protein while maintaining palatability. Significant price disparities are found across most gluten-free alternatives of gluten-containing foods due to this further processing requirement. In a study conducted by Missbach and colleagues [146], 63 gluten-free products and 126 of their gluten-containing counterparts were analyzed in 12 different Austrian supermarkets. The products included a broad range of items: bread, cereals, baking mixes, pasta, cookies, cakes, and snacks. Results showed that on average, gluten-free foods were 205% (cereals) to 267% (bread and bakery goods) more expensive than their gluten-containing counterparts. Whether this large price gap is because of overpricing due to high demand or processing costs is unclear. A 2-fold price gap between the two counterparts creates a tremendous burden on strict followers and may have detrimental financial effects. Other studies have confirmed this significant price difference as well [71,161]. Another study, conducted by Singh and Whelan [120], found that gluten-free products were more expensive (wheat-based products were 76–518% more expensive) and had limited availability in stores. Regular supermarkets had almost all the gluten-free alternative products (18/20, 90%); however, corner stores and budget supermarkets had limited gluten-free alternative products (1.8/20, 9%). Limited availability in convenience stores can further increase the cost of adherence, perhaps due to the time spent traveling to a regular supermarket that may be further away. One solution to circumvent this problem, provided by Di Nardo and colleagues [158], is to build the diet around naturally-occurring gluten-free foods and avoid the processed gluten-free alternatives altogether. This strategy can mitigate the price difference between the two counterparts and increase the number of stores one can buy from.

### 5.4. Social and Psychological Impact

Strict adherence to a gluten-free diet has been shown to cause some social and psychological adversities. Food is deeply embedded in cultures worldwide and can be found at the center of many social constructs. People gather and enjoy different foods to celebrate career accomplishments, weddings, religious rituals, and birthdays. Given that food exerts a significant influence on daily life, strict restrictions on dietary options can be a source of social isolation and unhappiness. In a study conducted by Zarkadas and colleagues [147], questionnaires were sent to members of the Canadian Celiac Association (5240 members) and 2681 adults (aged 16 or older) who had biopsy-proven CD. The questionnaire aimed to assess the recipients’ quality of life based on celiac-associated questions and the “SF-12,” a self-reported outcome measure assessing the impact of health on an individual’s everyday life. It was discovered that 44% of respondents reported having difficulties following the diet for various reasons, including determining if foods were gluten-free (85%), finding gluten-free foods in stores (83%), avoiding restaurants (79%), and avoiding travel (38%). However, due to the rising popularity of the gluten-free diet, many restaurants now include labels on the menu identifying any gluten-free items. Another study, conducted by Silvester et al. [148], further demonstrated the social isolation associated with the diet. The study found that non-CD responders to the questionnaire were less likely to adhere to the diet strictly and would sometimes ingest gluten intentionally. This group was associated with more pleasure and less anger and depression than CD responders who were stricter in adherence. The study also found that social isolation was more pronounced in CD responders, and eating was mainly at home instead of in public spaces. These results further demonstrate the challenges with adhering to the diet at the psychological and sociological levels. MacCulloch and Rashid [161] conducted a survey and found that improved labeling, government support through income, and education for schools and restaurants greatly help adherents of the diet. The social frustrations associated with a GFD can also be seen in type 1 diabetes, another autoimmune disease requiring restrictive dieting [162,163,164]. Patients with CD have shown a higher prevalence of type 1 diabetes mellitus than the general population (4.4–11.1% versus 0.5%), and Camarca and colleagues [165] found that 50% of patients with CD and type 1 diabetes comply with the GFD compared to the higher rate of 73% in patients with only CD. In adolescents, significantly, strict compliance has been associated with a worsened quality of life. Although not recognized by the Diagnostic and Statistical Manual of Mental Disorders, a phenomenon involving restrictive eating called orthorexia nervosa represents another cause for concern in adherents of the GFD. Orthorexia nervosa describes the behavior of healthy individuals who pursue increasingly restrictive diets despite not needing to do so (patients have a healthy weight and no diagnosed condition), and can experience a decrease in quality of life and overall health [166]. A study conducted by Wolf and colleagues [167] found that highly vigilant GFD adherents had lower quality of life due to anxiety, putting them at risk of orthorexia nervosa as they vigorously pursue their gluten-free lifestyle. It is crucial to address these socio-psychological issues as they tend to be harder to quantify. Close follow-up of quality of life, level of adherence with a GFD, and patient education on possible risks in CD patients following the diet is essential.

## 6. Conclusions

The GFD remains the primary treatment for celiac disease and may work in other health conditions. Patients with celiac disease must adhere to a lifelong GFD as it is currently the best-known treatment. Treatment of patients with celiac disease should be done at an early age, as younger individuals tend to show more significant reversal of gastrointestinal symptoms and healing from damage to the gut mucosa. While the diet is recognized in treating gluten ataxia, little is known about its other benefits. Patients with d-IBS and IBD experience relief in gastrointestinal symptoms following treatment with a GFD. Patients with NCSG experience similar improvements following the diet. Maintaining a strict gluten-free lifestyle has many challenges, including nutritional deficiencies, high costs due to adherence, and social and psychological barriers. These issues should be addressed when recommending the diet for any individual. More research is required to assess the benefits of the diet in treating mental, neurological, and cognitive diseases (depressive disorders, autism spectrum disorder, and “brain fog”, respectively). Large sample size studies can significantly help the current effort to assess the diet’s risks and benefits, which is needed to educate individuals who follow the diet without any diagnosis. This cohort of people makes up the most prominent GFD adherents who usually follow the diet because of the reported benefits. Studies that provide strong evidence are needed in order to aid individuals in making well-educated decisions on whether to follow the diet.

## Figures and Tables

**Table 1:** Modified Marsh-Oberhuber classification system.

Type	Intraepithelial Lymphocytes/100 Enterocytes (Duodenum)	Crypt	Villous Architecture	Lesion
0	<30	Normal	Normal	Pre-infiltrative
1	>30	Normal	Normal	Infiltrative
2	>30	Hyperplasia	Normal	Infiltrative-hyperplastic
3a	>30	Hyperplasia	Mild atrophy	Flat destructive
3b	>30	Hyperplasia	Marked atrophy	Flat destructive
3c	>30	Hyperplasia	Complete atrophy	Flat destructive

References [45,46,47].

## Data Availability

Not applicable.

## References

[1] Shiferaw B., Smale M., Braun H.-J., Duveiller E., Reynolds M., Muricho G. (2013). Crops that feed the world 10. Past successes and future challenges to the role played by wheat in global food security. Food Secur.

[2] Shurtleff W., Huang H.T., Aoyagi A. (2014). History of Soybeans and Soyfoods in China and Taiwan, and in Chinese Cookbooks, Restaurants, and Chinese Work with Soyfoods outside China (1024 BCE TO 2014).

[3] Nature or Nurture? Explaining English Wheat Yields in the Industrial Revolution, c.1770 on JSTOR. https://www.jstor.org/stable/3874947.

[4] Atack J., Margo R.A. (2011). The Impact of Access to Rail Transportation on Agricultural Improvement: The American Midwest as a Test Case, 1850–1860. J. Transp. Land Use.

[5] Fitzgerald D. (2020). World War II and the Quest for Time-Insensitive Foods. Osiris.

[6] Theien I. (2009). Food rationing during World War two: A special case of sustainable consumption?. Anthr. Food.

[7] Kearney J. (2010). Food consumption trends and drivers. Philos. Trans. R. Soc. B Biol. Sci..

[8] Wen S., Wen N., Pang J., Langen G., Brew-Appiah R., Mejías J., Osorio C.E., Yang M., Gemini R., Moehs C. (2012). Structural genes of wheat and barley 5-methylcytosine DNA glycosylases and their potential applications for human health. Proc. Natl. Acad. Sci. USA.

[9] Shewry P. (2019). What Is Gluten—Why Is It Special?. Front. Nutr..

[10] Schalk K., Lexhaller B., Koehler P., Scherf K.A. (2017). Isolation and characterization of gluten protein types from wheat, rye, barley and oats for use as reference materials. PLoS ONE.

[11] Balakireva A.V., Zamyatnin A.A. (2016). Properties of Gluten Intolerance: Gluten Structure, Evolution, Pathogenicity and Detoxification Capabilities. Nutrients.

[12] Li H., Wang J., Pan L., Lu Q. (2019). Effect of amino and thiol groups of wheat gluten on the quality characteristics of Chinese noodles. J. Food Sci. Technol..

[13] Moreno M.D.L., Rodríguez-Herrera A., Sousa C., Comino I. (2017). Biomarkers to Monitor Gluten-Free Diet Compliance in Celiac Patients. Nutrients.

[14] Gujral N. (2012). Celiac disease: Prevalence, diagnosis, pathogenesis and treatment. World J. Gastroenterol..

[15] Caio G., Volta U., Sapone A., Leffler D.A., de Giorgio R., Catassi C., Fasano A. (2019). Celiac disease: A comprehensive current review. BMC Med..

[16] Stazi A.V., Trecca A., Trinti B. (2008). Osteoporosis in celiac disease and in endocrine and reproductive disorders. World J. Gastroenterol..

[17] Ndez-Bañares H.M., Monzón H., Forné M. (2009). A short review of malabsorption and anemia. World J. Gastroenterol..

[18] Rondanelli M., Faliva M.A., Gasparri C., Peroni G., Naso M., Picciotto G., Riva A., Nichetti M., Infantino V., Alalwan T. (2019). Micronutrients Dietary Supplementation Advices for Celiac Patients on Long-Term Gluten-Free Diet with Good Compliance: A Review. Medicina.

[19] Kreutz J.M., Adriaanse M.P.M., Van Der Ploeg E.M.C., Vreugdenhil A.C.E. (2020). Narrative Review: Nutrient Deficiencies in Adults and Children with Treated and Untreated Celiac Disease. Nutrients.

[20] Barker J.M., Liu E. (2008). Celiac Disease: Pathophysiology, Clinical Manifestations, and Associated Autoimmune Conditions. Adv. Pediatr..

[21] Balaban D.V., Dima A., Jurcut C., Popp A., Jinga M. (2019). Celiac crisis, a rare occurrence in adult celiac disease: A systematic review. World J. Clin. Cases.

[22] De Pablo P., Cooper M.S., Buckley C.D. (2012). Association between bone mineral density and C-reactive protein in a large population-based sample. Arthritis Rheum..

[23] Hardy R., Cooper M.S. (2009). Bone loss in inflammatory disorders. J. Endocrinol..

[24] Khundmiri S.J., Murray R.D., Lederer E. (2016). PTH and Vitamin D. Compr. Physiol..

[25] Flynn A. (2003). The role of dietary calcium in bone health. Proc. Nutr. Soc..

[26] Van Rijn J.C.W., Grote F.K., Oostdijk W., Wit J.M. (2004). Short stature and the probability of coeliac disease, in the absence of gastrointestinal symptoms. Arch. Dis. Child..

[27] Garganta M.D., Bremer A.A. (2014). Clinical Dilemmas in Evaluating the Short Child. Pediatr. Ann..

[28] Ludvigsson J.F., Michaelsson K., Ekbom A., Montgomery S.M. (2006). Coeliac disease and the risk of fractures—A general population-based cohort study. Aliment. Pharmacol. Ther..

[29] Melton L.J., Beck T.J., Amin S., Khosla S., Achenbach S.J., Oberg A.L., Riggs B.L. (2005). Contributions of bone density and structure to fracture risk assessment in men and women. Osteoporos. Int..

[30] Kemppainen T., Kröger H., Janatuinen E., Arnala I., Kosma V.-M., Pikkarainen P., Julkunen R., Jurvelin J., Alhava E., Uusitupa M. (1999). Osteoporosis in adult patients with celiac disease. Bone.

[31] Sollid L.M., McAdam S.N., Molberg Á., Quarsten H., Arentz-Hansen H., Louka A.S., Lundin K.E.A. (2001). Genes and envi-ronment in celiac disease. Acta Odontol. Scand..

[32] Lionetti E., Catassi C. (2015). The Role of Environmental Factors in the Development of Celiac Disease: What Is New?. Diseases.

[33] Tian N., Leffler D.A., Kelly C.P., Hansen J., Marietta E.V., Murray J.A., Schuppan D., Helmerhorst E.J. (2015). Despite sequence homologies to gluten, salivary proline-rich proteins do not elicit immune responses central to the pathogenesis of celiac disease. Am. J. Physiol. Liver Physiol..

[34] Fernández-Pérez S., Pérez-Andrés J., Gutiérrez S., Navasa N., Martínez-Blanco H., Ferrero M., Vivas S., Vaquero L., Iglesias C., Casqueiro J. (2020). The Human Digestive Tract Is Capable of Degrading Gluten from Birth. Int. J. Mol. Sci..

[35] Camarca A., Anderson R.P., Mamone G., Fierro O., Facchiano A., Costantini S., Zanzi D., Sidney J., Auricchio S., Sette A. (2009). Intestinal T Cell Responses to Gluten Peptides Are Largely Heterogeneous: Implications for a Peptide-Based Therapy in Celiac Disease. J. Immunol..

[36] Drago S., El Asmar R., Di Pierro M., Clemente M.G., Sapone A.T.A., Thakar M., Iacono G., Carroccio A., D’Agate C., Not T. (2006). Gliadin, zonulin and gut permeability: Effects on celiac and non-celiac intestinal mucosa and intestinal cell lines. Scand. J. Gastroenterol..

[37] Clemente M.G., De Virgiliis S., Kang J.S., Macatagney R., Musu M.P., Di Pierro M.R., Drago S., Congia M., Fasano A. (2003). Early effects of gliadin on enterocyte intracellular signalling involved in intestinal barrier function. Gut.

[38] Fasano A. (2012). Zonulin, regulation of tight junctions, and autoimmune diseases. Ann. N. Y. Acad. Sci..

[39] Ráki M., Schjetne K.W., Stamnaes J., Molberg Ø., Jahnsen F.L., Issekutz T.B., Bogen B., Sollid L.M. (2007). Surface Expression of Transglutaminase 2 by Dendritic Cells and its Potential Role for Uptake and Presentation of Gluten Peptides to T Cells. Scand. J. Immunol..

[40] Cecilio L.A., Bonatto M.W. (2015). The Prevalence Of Hla Dq2 And Dq8 In Patients With Celiac Disease, In Family and in General Population. Arq. Bras. Cir. Dig..

[41] Mazzarella G. (2015). Effector and suppressor T cells in celiac disease. World J. Gastroenterol..

[42] La Scaleia R., Barba M., Di Nardo G., Bonamico M., Oliva S., Nenna R., Valitutti F., Mennini M., Barbato M., Montuori M. (2012). Size and dynamics of mucosal and peripheral IL-17A+ T-cell pools in pediatric age, and their disturbance in celiac disease. Mucosal Immunol..

[43] Parzanese I., Qehajaj D., Patrinicola F., Aralica M., Chiriva-Internati M., Stifter S., Elli L., Grizzi F. (2017). Celiac disease: From pathophysiology to treatment. World J. Gastrointest. Pathophysiol..

[44] Ludvigsson J.F., Bai J.C., Biagi F., Card T., Ciacci C., Ciclitira P.J., Green P.H.R., Hadjivassiliou M., Holdoway A., van Heel D. (2014). Diagnosis and management of adult coeliac disease: Guidelines from the British Society of Gastroenterology. Gut.

[45] Corazza G.R. (2005). Coeliac disease. J. Clin. Pathol..

[46] Bañares F.F., Mariné M., Rosinach M., Carrasco A., Esteve M., Rodrigo L. (2014). Type 1 Marsh Celiac Disease: Diagnosis and Response. OmniaScience Monogr..

[47] Kamboj A.K., Oxentenko A.S. (2017). Clinical and Histologic Mimickers of Celiac Disease. Clin. Transl. Gastroenterol..

[48] Itzlinger A., Branchi F., Elli L., Schumann M. (2018). Gluten-Free Diet in Celiac Disease—Forever and for All?. Nutrients.

[49] Green P.H., Fleischauer A.T., Bhagat G., Goyal R., Jabri B., Neugut A.I. (2003). Risk of malignancy in patients with celiac disease. Am. J. Med..

[50] Niland B., Cash B.D. (2018). Health Benefits and Adverse Effects of a Gluten-Free Diet in Non–Celiac Disease Patients. Gastroenterol. Hepatol..

[51] Valitutti F., Iorfida D., Anania C., Trovato C.M., Montuori M., Cucchiara S., Catassi C. (2017). Cereal Consumption among Subjects with Celiac Disease: A Snapshot for Nutritional Considerations. Nutrients.

[52] Jones A.L. (2017). The Gluten-Free Diet: Fad or Necessity?. Diabetes Spectr..

[53] Wahab P.J., Meijer J.W., Mulder C.J. (2002). Histologic Follow-up of People With Celiac Disease on a Gluten-Free Diet. Am. J. Clin. Pathol..

[54] Rubio-Tapia A., Rahim M.W., See J.A., Lahr B.D., Wu T.-T., Murray J.A. (2010). Mucosal Recovery and Mortality in Adults With Celiac Disease After Treatment With a Gluten-Free Diet. Am. J. Gastroenterol..

[55] Tursi A., Brandimarte G., Giorgetti G., Elisei W., Inchingolo C., Monardo E., Aiello F. (2006). Endoscopic and histological findings in the duodenum of adults with celiac disease before and after changing to a gluten-free diet: A 2-year prospective study. Endoscopy.

[56] Kavak U.S., Yüce A., Koçak N., Demir H., Saltik I.N., Gürakan F., Özen H. (2003). Bone Mineral Density in Children With Untreated and Treated Celiac Disease. J. Pediatr. Gastroenterol. Nutr..

[57] Nicoletta M., Maria Pia C., Maria Teresa B., Sergio O., Giorgio Giambattista G., Paolo B. (1990). Bone Mineral Density in Adult Celiac Patients and the Effect of Gluten-Free Diet from Childhood. Am. J. Gastroenterol..

[58] Barera G., Mora S., Brambilla P., Ricotti A., Menni L., Beccio S., Bianchi C. (2000). Body composition in children with celiac disease and the effects of a gluten-free diet: A prospective case-control study. Am. J. Clin. Nutr..

[59] Kalayci A.G., Kansu A., Girgin N., Kucuk N.O., Aras G. (2001). Bone Mineral Density and Importance of a Gluten-Free Diet in Patients With Celiac Disease in Childhood. Pediatrics.

[60] Soliman A.T., Laham M., Jour C., Shaat M., Souikey F., Itani M., Al-Safi A., Karmallah A., Qudaisat A., Alarabi Z. (2019). Linear growth of children with celiac disease after the first two years on gluten- free diet: A controlled study. Acta Biomed.

[61] Kurppa K., Collin P., Viljamaa M., Haimila K., Saavalainen P., Partanen J., Laurila K., Huhtala H., Paasikivi K., Mäki M. (2009). Diagnosing Mild Enteropathy Celiac Disease: A Randomized, Controlled Clinical Study. Gastroenterology.

[62] Tursi A., Brandimarte G. (2003). The Symptomatic and Histologic Response to a Gluten-Free Die in Patients With Borderline Enteropathy. J. Clin. Gastroenterol..

[63] Wieser H., Segura V., Ruiz-Carnicer A., Sousa C., Comino I. (2021). Food Safety and Cross-Contamination of Gluten-Free Products: A Narrative Review. Nutrients.

[64] Hischenhuber C., Crevel R., Jarry B., Maki M., Moneret-Vautrin D.A., Romano A., Troncone R., Ward R. (2006). Review article: Safe amounts of gluten for patients with wheat allergy or coeliac disease. Aliment. Pharmacol. Ther..

[65] Lähdeaho M.-L., Mäki M., Laurila K., Huhtala H., Kaukinen K. (2011). Small- bowel mucosal changes and antibody responses after low- and moderate-dose gluten challenge in celiac disease. BMC Gastroenterol..

[66] Akobeng A.K., Thomas A.G. (2008). Systematic review: Tolerable amount of gluten for people with coeliac disease. Aliment. Pharmacol. Ther..

[67] Catassi C., Rossini M., Ratsch I.M., Bearzi I., Santinelli A., Castagnani R., Pisani E., Coppa G.V., Giorgi P.L. (1993). Dose dependent effects of protracted ingestion of small amounts of gliadin in coeliac disease children: A clinical and jejunal morphometric study. Gut.

[68] Dunne M., Byrne G., Chirdo F.G., Feighery C. (2020). Coeliac Disease Pathogenesis: The Uncertainties of a Well-Known Immune Mediated Disorder. Front. Immunol..

[69] Lerner B.A., Vo L.T.P., Yates S., Rundle A.G., Green P.H., Lebwohl B. (2019). Detection of Gluten in Gluten-Free Labeled Restaurant Food: Analysis of Crowd-Sourced Data. Am. J. Gastroenterol..

[70] Collin P., Thorell L., Kaukinen K., Mäki M. (2004). The safe threshold for gluten contamination in gluten-free products. Can trace amounts be accepted in the treatment of coeliac disease?. Aliment. Pharmacol. Ther..

[71] Stevens L., Rashid M. (2008). Gluten-Free and Regular Foods: A Cost Comparison. Can. J. Diet. Pr. Res..

[72] Lee A.R., Ng D.L., Zivin J., Green P.H.R. (2007). Economic burden of a gluten-free diet. J. Hum. Nutr. Diet..

[73] Singh J., Whelan K. (2011). Limited availability and higher cost of gluten-free foods. J. Hum. Nutr. Diet..

[74] Mustalahti K., Lohiniemi S., Collin P., Vuolteenaho N., Laippala P., Mäki M. (2002). Gluten-free diet and quality of life in patients with screen-detected celiac disease. Eff. Clin. Pract..

[75] Plugis N.M., Khosla C. (2015). Therapeutic approaches for celiac disease. Best Pr. Res. Clin. Gastroenterol..

[76] Bebb J.R., Lawson A., Knight T., Long R.G. (2006). Long-term follow-up of coeliac disease—What do coeliac patients want?. Aliment. Pharmacol. Ther..

[77] Kurppa K., Lauronen O., Collin P., Ukkola A., Laurila K., Huhtala H., Mäki M., Kaukinen K. (2012). Factors Associated with Dietary Adherence in Celiac Disease: A Nationwide Study. Digestion.

[78] Galli G., Carabotti M., Pilozzi E., Lahner E., Annibale B., Conti L. (2021). Relationship between Persistent Gastrointestinal Symptoms and Duodenal Histological Findings after Adequate Gluten-Free Diet: A Gray Area of Celiac Disease Management in Adult Patients. Nutrients.

[79] Leffler D.A., Dennis M., George J.E., Jamma S., Cook E.F., Schuppan D., Kelly C.P. (2009). A Validated Disease-Specific Symptom Index for Adults With Celiac Disease. Clin. Gastroenterol. Hepatol..

[80] Leffler D.A., Dennis M., George J.B.E., Jamma S., Magge S., Cook E.F., Schuppan D., Kelly C.P. (2009). A Simple Validated Gluten-Free Diet Adherence Survey for Adults With Celiac Disease. Clin. Gastroenterol. Hepatol..

[81] Gładyś K., Dardzińska J., Guzek M., Adrych K., Małgorzewicz S. (2020). Celiac Dietary Adherence Test and Standardized Dietician Evaluation in Assessment of Adherence to a Gluten-Free Diet in Patients with Celiac Disease. Nutrients.

[82] Wieser H., Ruiz-Carnicer Á., Segura V., Comino I., Sousa C. (2021). Challenges of Monitoring the Gluten-Free Diet Adherence in the Management and Follow-Up of Patients with Celiac Disease. Nutrients.

[83] Hill I.D. (2005). What are the sensitivity and specificity of serologic tests for celiac disease? Do sensitivity and specificity vary in different populations?. Gastroenterology.

[84] Rostom A., Dubé C., Cranney A., Saloojee N., Sy R., Garritty C., Sampson M., Zhang L., Yazdi F., Mamaladze V. (2005). The diagnostic accuracy of serologic tests for celiac disease: A systematic review. Gastroenterology.

[85] Maglione M.A., Okunogbe A., Ewing B., Grant S., Newberry S.J., Motala A., Shanman R., Mejia N., Arifkhanova A., Shekelle P. (2016). Diagnosis of Celiac Disease. Diagnosis Celiac Dis..

[86] Vitoria J.C., Arrieta A., Arranz C., Ayesta A., Sojo A., Maruri N., García-Masdevall M.D. (1999). Antibodies to Gliadin, Endomysium, and Tissue Transglutaminase for the Diagnosis of Celiac Disease. J. Pediatr. Gastroenterol. Nutr..

[87] Maki M., Holm K., Hallstrom O., Collin P., Viander M., Savilahti E., Lipsanen V., Koskimies S. (1991). Serological markers and HLA genes among healthy first-degree relatives of patients with coeliac disease. Lancet.

[88] Adriaanse M., Leffler D.A. (2015). Serum Markers in the Clinical Management of Celiac Disease. Dig. Dis..

[89] Lewis N.R., Scott B.B. (2010). Meta-analysis: Deamidated gliadin peptide antibody and tissue transglutaminase antibody compared as screening tests for coeliac disease. Aliment. Pharmacol. Ther..

[90] Barbato M., Maiella G., Di Camillo C., Guida S., Valitutti F., Lastrucci G., Mainiero F., Cucchiara S. (2011). The anti-deamidated gliadin peptide antibodies unmask celiac disease in small children with chronic diarrhoea. Dig. Liver Dis..

[91] Monzani A., Rapa A., Fonio P., Tognato E., Panigati L., Oderda G. (2011). Use of Deamidated Gliadin Peptide Antibodies to Monitor Diet Compliance in Childhood Celiac Disease. J. Pediatr. Gastroenterol. Nutr..

[92] Liu E., Li M., Emery L., Taki I., Barriga K., Tiberti C., Eisenbarth G.S., Rewers M.J., Hoffenberg E.J. (2007). Natural History of Antibodies to Deamidated Gliadin Peptides and Transglutaminase in Early Childhood Celiac Disease. J. Pediatr. Gastroenterol. Nutr..

[93] Silvester J.A., Kurada S., Szwajcer A., Kelly C.P., Leffler D.A., Duerksen D.R. (2017). Tests for Serum Transglutaminase and Endomysial Antibodies Do Not Detect Most Patients With Celiac Disease and Persistent Villous Atrophy on Gluten-free Diets: A Meta-analysis. Gastroenterology.

[94] Comino I., Real A., Vivas S., Síglez M., Caminero A., Nistal E., Casqueiro J., Rodríguez-Herrera A., Cebolla Á., Sousa C. (2012). Monitoring of gluten-free diet compliance in celiac patients by assessment of gliadin 33-mer equivalent epitopes in feces. Am. J. Clin. Nutr..

[95] Sonnenburg J.L., Fischbach M.A. (2011). Community Health Care: Therapeutic Opportunities in the Human Microbiome. Sci. Transl. Med..

[96] Morón B., Cebolla Á., Manyani H., Álvarez-Maqueda M., Megías M., Thomas M.D.C., López M.C., Sousa C. (2008). Sensitive detection of cereal fractions that are toxic to celiac disease patients by using monoclonal antibodies to a main immunogenic wheat peptide. Am. J. Clin. Nutr..

[97] Comino I., Segura V., Ortigosa L., Espín B., Castillejo G., Garrote J.A., Sierra C., Millán-Jiménez A., Ribes-Koninckx C., Román E. (2019). Prospective longitudinal study: Use of faecal gluten immunogenic peptides to monitor children diagnosed with coeliac disease during transition to a gluten-free diet. Aliment. Pharmacol. Ther..

[98] Cebolla Á., Moreno M.D.L., Coto L., Sousa C. (2018). Gluten Immunogenic Peptides as Standard for the Evaluation of Potential Harmful Prolamin Content in Food and Human Specimen. Nutrients.

[99] Baviera L.C.B., Aliaga E.D., Ortigosa L., Litwin N., Peña-Quintana L., Méndez V., González M.V., López-Manzanares J.M., Méndez E., Koninckx C.R. (2007). Celiac Disease Screening by Immunochromatographic Visual Assays: Results of a Multicenter Study. J. Pediatr. Gastroenterol. Nutr..

[100] Comino I., Fernández-Bañares F., Esteve M., Ortigosa L., Castillejo G., Fambuena B., Ribes-Koninckx C., Sierra C., Rodríguez-Herrera A., Salazar J.C. (2016). Fecal Gluten Peptides Reveal Limitations of Serological Tests and Food Questionnaires for Monitoring Gluten-Free Diet in Celiac Disease Patients. Am. J. Gastroenterol..

[101] Moreno M.D.L., Cebolla Á., Muñoz-Suano A., Carrillo-Carrion C., Comino I., Pizarro Á., León F., Rodríguez-Herrera A., Sousa C. (2015). Detection of gluten immunogenic peptides in the urine of patients with coeliac disease reveals transgressions in the gluten-free diet and incomplete mucosal healing. Gut.

[102] Leonard M.M., Weir D.C., DeGroote M., Mitchell P.D., Singh P., Silvester J., Leichtner A.M., Fasano A. (2017). Value of IgA tTG in Predicting Mucosal Recovery in Children With Celiac Disease on a Gluten-Free Diet. J. Pediatr. Gastroenterol. Nutr..

[103] Pais W.P., Duerksen D.R., Pettigrew N.M., Bernstein C.N. (2008). How many duodenal biopsy specimens are required to make a diagnosis of celiac disease?. Gastrointest. Endosc..

[104] Rubio-Tapia A., Hill I.D., Kelly C.P., Calderwood A., Murray J.A. (2013). ACG Clinical Guidelines: Diagnosis and Management of Celiac Disease. Am. J. Gastroenterol..

[105] Husby S., Koletzko S., Korponay-Szabó I., Mearin M., Phillips A., Shamir R., Troncone R., Giersiepen K., Branski D., Catassi C. (2012). European Society for Pediatric Gastroenterology, Hepatology, and Nutrition Guidelines for the Diagnosis of Coeliac Disease. J. Pediatr. Gastroenterol. Nutr..

[106] Choung R.S., Unalp-Arida A., Ruhl C.E., Brantner T.L., Everhart J.E., Murray J.A. (2016). Less Hidden Celiac Disease But Increased Gluten Avoidance Without a Diagnosis in the United States. Mayo Clin. Proc..

[107] Hadjivassiliou M., Aeschlimann P., Sanders D.S., Mäki M., Kaukinen K., Grunewald R.A., Bandmann O., Woodroofe N., Haddock G., Aeschlimann D. (2013). Transglutaminase 6 antibodies in the diagnosis of gluten ataxia. Neurology.

[108] Dipper C.R., Maitra S., Thomas R., Lamb C.A., Mclean-Tooke A.P.C., Ward R., Smith D., Spickett G., Mansfield J.C. (2009). Anti-tissue transglutaminase antibodies in the follow-up of adult coeliac disease. Aliment. Pharmacol. Ther..

[109] Hadjivassiliou M., Gibson A., Davies-Jones G., Lobo A., Stephenson T., Milford-Ward A. (1996). Does cryptic gluten sensitivity play a part in neurological illness?. Lancet.

[110] Buie T. (2013). The Relationship of Autism and Gluten. Clin. Ther..

[111] Lau N.M., Green P.H.R., Taylor A.K., Hellberg D., Ajamian M., Tan C.Z., Kosofsky B.E., Higgins J.J., Rajadhyaksha A.M., Alaedini A. (2013). Markers of Celiac Disease and Gluten Sensitivity in Children with Autism. PLoS ONE.

[112] Ghalichi F., Ghaemmaghami J., Malek A., Ostadrahimi A. (2016). Effect of gluten free diet on gastrointestinal and behavioral indices for children with autism spectrum disorders: A randomized clinical trial. World J. Pediatr..

[113] Piwowarczyk A., Horvath A., Pisula E., Kawa R., Szajewska H. (2019). Gluten-Free Diet in Children with Autism Spectrum Disorders: A Randomized, Controlled, Single-Blinded Trial. J. Autism Dev. Disord..

[114] Peters S.L., Biesiekierski J., Yelland G., Muir J.G., Gibson P.R. (2014). Randomised clinical trial: Gluten may cause depression in subjects with non-coeliac gluten sensitivity—An exploratory clinical study. Aliment. Pharmacol. Ther..

[115] Zylberberg H.M., Demmer R.T., Murray J.A., Green P.H., Lebwohl B. (2017). Depression and insomnia among individuals with celiac disease or on a gluten-free diet in the USA. Eur. J. Gastroenterol. Hepatol..

[116] Ohrnberger J., Fichera E., Sutton M. (2017). The relationship between physical and mental health: A mediation analysis. Soc. Sci. Med..

[117] Blanchet S., Chikhi S., Maltais D. (2018). The benefits of physical activities on cognitive and mental health in healthy and pathological aging. Geriatrie et psychologie neuropsychiatrie du vieillissement.

[118] Ergün C., Urhan M., Ayer A. (2017). A review on the relationship between gluten and schizophrenia: Is gluten the cause?. Nutr. Neurosci..

[119] Levinta A., Mukovozov I., Tsoutsoulas C. (2018). Use of a Gluten-Free Diet in Schizophrenia: A Systematic Review. Adv. Nutr..

[120] Wahnschaffe U., Ullrich R., Riecken E., Schulzke J. (2001). Celiac disease–like abnormalities in a subgroup of patients with irritable bowel syndrome. Gastroenterology.

[121] Aziz I., Trott N., Briggs R., North J.R., Hadjivassiliou M., Sanders D.S. (2015). Efficacy of a Gluten-Free Diet in Subjects With Irritable Bowel Syndrome-Diarrhea Unaware of Their HLA-DQ2/8 Genotype. Clin. Gastroenterol. Hepatol..

[122] (2015). Celiac disease, non-celiac gluten sensitivity and inflammatory bowel disease. Minerva Gastroenterol. Dietol..

[123] Herfarth H.H., Martin C.F., Sandler R.S., Kappelman M.D., Long M.D. (2014). Prevalence of a Gluten-free Diet and Improvement of Clinical Symptoms in Patients with Inflammatory Bowel Diseases. Inflamm. Bowel Dis..

[124] Lindberg E., Magnusson K.-E., Tysk C., Jarnerot G. (1992). Antibody (IgG, IgA, and IgM) to baker’s yeast (Saccharomyces cerevisiae), yeast mannan, gliadin, ovalbumin and betalactoglobulin in monozygotic twins with inflammatory bowel disease. Gut.

[125] Reunala T., Blomqvist K., Tarpila S., Halme H., Kangas K. (1977). Gluten-free diet in dermatitis herpetiformis. Br. J. Dermatol..

[126] Fry L., Riches D., Seah P., Hoffbrand A. (1973). Clearance of skin lesions in dermatitis herpetiformis after gluten withdrawal. Lancet.

[127] Catassi C., Elli L., Bonaz B., Bouma G., Carroccio A., Castillejo G., Cellier C., Cristofori F., De Magistris L., Dolinsek J. (2015). Diagnosis of Non-Celiac Gluten Sensitivity (NCGS): The Salerno Experts’ Criteria. Nutrients.

[128] Kabbani T.A., Vanga R.R., Leffler D.A., Villafuerte-Galvez J., Pallav K., Hansen J., Mukherjee R., Dennis M., Kelly C.P. (2014). Celiac Disease or Non-Celiac Gluten Sensitivity? An Approach to Clinical Differential Diagnosis. Am. J. Gastroenterol..

[129] Roszkowska A., Pawlicka M., Mroczek A., Bałabuszek K., Nieradko-Iwanicka B. (2019). Non-Celiac Gluten Sensitivity: A Review. Medicina.

[130] Fasano A., Sapone A., Zevallos V., Schuppan D. (2015). Nonceliac Gluten Sensitivity. Gastroenterology.

[131] Pellegrina C.D., Perbellini O., Scupoli M., Tomelleri C., Zanetti C., Zoccatelli G., Fusi M., Peruffo A., Rizzi C., Chignola R. (2009). Effects of wheat germ agglutinin on human gastrointestinal epithelium: Insights from an experimental model of immune/epithelial cell interaction. Toxicol. Appl. Pharmacol..

[132] Skodje G.I., Sarna V.K., Minelle I.H., Rolfsen K.L., Muir J.G., Gibson P.R., Veierød M.B., Henriksen C., Lundin K.E. (2018). Fructan, Rather Than Gluten, Induces Symptoms in Patients With Self-Reported Non-Celiac Gluten Sensitivity. Gastroenterology.

[133] Yelland G.W. (2017). Gluten-induced cognitive impairment (“brain fog”) in coeliac disease. J. Gastroenterol. Hepatol..

[134] Truswell A.S. (2002). Cereal grains and coronary heart disease. Eur. J. Clin. Nutr..

[135] Flight I., Clifton P. (2006). Cereal grains and legumes in the prevention of coronary heart disease and stroke: A review of the literature. Eur. J. Clin. Nutr..

[136] Jensen M.K., Koh-Banerjee P., Hu F.B., Franz M., Sampson L., Grønbaek M., Rimm E.B. (2004). Intakes of whole grains, bran, and germ and the risk of coronary heart disease in men. Am. J. Clin. Nutr..

[137] Tang G., Wang D., Long J., Yang F., Si L. (2015). Meta-Analysis of the Association Between Whole Grain Intake and Coronary Heart Disease Risk. Am. J. Cardiol..

[138] Lebwohl B., Cao Y., Zong G., Hu F.B., Green P.H.R., Neugut A.I., Rimm E.B., Sampson L., Dougherty L.W., Giovannucci E. (2017). Long term gluten consumption in adults without celiac disease and risk of coronary heart disease: Prospective cohort study. BMJ.

[139] Potter M.D.E., Brienesse S.C., Walker M.M., Boyle A., Talley N.J. (2017). Effect of the gluten-free diet on cardiovascular risk factors in patients with coeliac disease: A systematic review. J. Gastroenterol. Hepatol..

[140] Heikkilä K., Koskinen O., Agarwal A., Tikkinen K., Mäki M., Kaukinen K. (2015). Associations of coeliac disease with coronary heart disease and cerebrovascular disease: A systematic review and meta-analysis. Nutr. Metab. Cardiovasc. Dis..

[141] Kim H.-S., Demyen M.F., Mathew J., Kothari N., Feurdean M., Ahlawat S.K. (2017). Obesity, Metabolic Syndrome, and Cardiovascular Risk in Gluten-Free Followers Without Celiac Disease in the United States: Results from the National Health and Nutrition Examination Survey 2009–2014. Dig. Dis. Sci..

[142] Deora V., Aylward N., Sokoro A., El-Matary W. (2017). Serum Vitamins and Minerals at Diagnosis and Follow-up in Children With Celiac Disease. J. Pediatr. Gastroenterol. Nutr..

[143] Thompson T., Dennis M., Higgins L.A., Lee A.R., Sharrett M.K. (2005). Gluten-free diet survey: Are Americans with coeliac disease consuming recommended amounts of fibre, iron, calcium and grain foods?. J. Hum. Nutr. Diet..

[144] Sue A., Dehlsen K., Ooi C.Y. (2018). Paediatric Patients with Coeliac Disease on a Gluten-Free Diet: Nutritional Adequacy and Macro- and Micronutrient Imbalances. Curr. Gastroenterol. Rep..

[145] Hemarajata P., Versalovic J. (2012). Effects of probiotics on gut microbiota: Mechanisms of intestinal immunomodulation and neuromodulation. Ther. Adv. Gastroenterol..

[146] Missbach B., Schwingshackl L., Billmann A., Mystek A., Hickelsberger M., Bauer G., Koenig J. (2015). Gluten-free food database: The nutritional quality and cost of packaged gluten-free foods. PeerJ.

[147] Zarkadas M., Cranney A., Case S., Molloy M., Switzer C., Graham I.D., Butzner J.D., Rashid M., Warren R.E., Burrows V. (2006). The impact of a gluten-free diet on adults with coeliac disease: Results of a national survey. J. Hum. Nutr. Diet..

[148] Silvester J.A., Weiten D., Graff L.A., Walker J.R., Duerksen D.R. (2015). Living gluten-free: Adherence, knowledge, lifestyle adaptations and feelings towards a gluten-free diet. J. Hum. Nutr. Diet..

[149] Jandhyala S.M. (2015). Role of the normal gut microbiota. World J. Gastroenterol..

[150] Dominguez-Bello M.G., Costello E.K., Contreras M., Magris M., Hidalgo G., Fierer N., Knight R. (2010). Delivery mode shapes the acquisition and structure of the initial microbiota across multiple body habitats in newborns. Proc. Natl. Acad. Sci. USA.

[151] Bohnhoff M., Miller C.P. (1962). Enhanced Susceptibility to Salmonella Infection in Streptomycin-Treated Mice. J. Infect. Dis..

[152] David L.A., Maurice C.F., Carmody R.N., Gootenberg D., Button J.E., Wolfe B.E., Ling A.V., Devlin A.S., Varma Y., Fischbach M. (2013). Diet rapidly and reproducibly alters the human gut microbiome. Nature.

[153] Golfetto L., De Senna F.D., Hermes J., Beserra B.T.S., França F.D.S., Martinello F. (2014). Lower bifidobacteria counts in adult patients with celiac disease on a gluten-free diet. Arq. Gastroenterol..

[154] Wacklin P., Laurikka P., Lindfors K., Collin P., Salmi T., Lähdeaho M.-L., Saavalainen P., Mäki M., Mättö J., Kurppa K. (2014). Altered Duodenal Microbiota Composition in Celiac Disease Patients Suffering From Persistent Symptoms on a Long-Term Gluten-Free Diet. Am. J. Gastroenterol..

[155] De Palma G., Nadal I., Collado M.C., Sanz Y. (2009). Effects of a gluten-free diet on gut microbiota and immune function in healthy adult human subjects. Br. J. Nutr..

[156] Yang B., Xiao L., Liu S., Liu X., Luo Y., Ji Q., Yang P., Liu Z. (2017). Exploration of the effect of probiotics supplementation on intestinal microbiota of food allergic mice. Am. J. Transl. Res..

[157] Sánchez B., Delgado S., Blanco-Míguez A., Lourenço A., Gueimonde M., Margolles A. (2016). Probiotics, gut microbiota, and their influence on host health and disease. Mol. Nutr. Food Res..

[158] Di Nardo G., Villa M.P., Conti L., Ranucci G., Pacchiarotti C., Principessa L., Raucci U., Parisi P. (2019). Nutritional Deficiencies in Children with Celiac Disease Resulting from a Gluten-Free Diet: A Systematic Review. Nutrients.

[159] Mariani P., Viti M.G., Montouri M., La Vecchia A., Cipolletta E., Calvani L., Bonamico M. (1998). The Gluten-Free Diet: A Nutritional Risk Factor for Adolescents with Celiac Disease?. J. Pediatr. Gastroenterol. Nutr..

[160] Caponio F., Summo C., Clodoveo M.L., Pasqualone A. (2007). Evaluation of the nutritional quality of the lipid fraction of gluten-free biscuits. Eur. Food Res. Technol..

[161] MacCulloch K., Rashid M. (2014). Factors affecting adherence to a gluten-free diet in children with celiac disease. Paediatr. Child Heal..

[162] Lennerz B.S., Barton A., Bernstein R.K., Dikeman R.D., Diulus C., Hallberg S., Rhodes E.T., Ebbeling C.B., Westman E.C., Yancy W.S. (2018). Management of Type 1 Diabetes With a Very Low–Carbohydrate Diet. Pediatrics.

[163] Rosenfalck A.M., Almdal T., Viggers L., Madsbad S., Hilsted J. (2006). A low-fat diet improves peripheral insulin sensitivity in patients with Type 1 diabetes. Diabet. Med..

[164] Patton S.R. (2011). Adherence to Diet in Youth with Type 1 Diabetes. J. Am. Diet. Assoc..

[165] Camarca M.E., Mozzillo E., Nugnes R., Zito E., Falco M., Fattorusso V., Mobilia S., Buono P., Valerio G., Troncone R. (2012). Celiac disease in type 1 diabetes mellitus. Ital. J. Pediatrics.

[166] Scarff J.R. (2017). Orthorexia Nervosa: An Obsession With Healthy Eating. Fed. Pract..

[167] Wolf R.L., Lebwohl B., Lee A.R., Zybert P., Reilly N.R., Cadenhead J., Amengual C., Green P.H.R. (2018). Hypervigilance to a Gluten-Free Diet and Decreased Quality of Life in Teenagers and Adults with Celiac Disease. Dig. Dis. Sci..

